# Young Learners’ Regulation of Practice Behavior in Adaptive Learning Technologies

**DOI:** 10.3389/fpsyg.2019.02792

**Published:** 2019-12-13

**Authors:** Inge Molenaar, Anne Horvers, Rick Dijkstra

**Affiliations:** Behavioural Science Institute, Radboud University, Nijmegen, Netherlands

**Keywords:** self-regulated learning, Adaptive Learning Technologies, self-evaluation, calibration, primary education

## Abstract

Although research indicates positive effects of Adaptive Learning Technologies (ALTs) on learning, we know little about young learners’ regulation intentions in this context. Learners’ intentions and self-evaluation determine the signals they deduce to drive self-regulated learning. This study had a twofold approach as it investigated the effect of feed-up and feed-forward reports on practice behavior and learning and explored learners’ self-evaluation of goal-attainment, performance and accuracy. In the experimental condition, learners described their goals and self-evaluated their progress in feed-up and forward reports. We found no conclusive effects of the feed-up and forward reports on learners’ regulation of practice behavior and learning. Furthermore, results indicated that young learners’ self-evaluations of goal attainment and performance were biased. Contrary to other research, we found learners both over- and underestimated performance which was strongly associated with over- or underestimation of goal attainment. Hence the signals learners used to drive regulation were often incorrect, tending to induce over- or under-practicing. Similarly, we found a bias in self-evaluation of accuracy and accuracy attainment. Learners over- or underestimated their accuracy, which was associated with over- or underestimation of accuracy attainment, which may in turn have affected effort regulation. We concluded that goal setting and self-evaluation in feed-up and forward reports was not enough to deduce valid regulatory signals. Our results indicate that young learners needed performance feedback to support correct self-evaluation and to correctly drive regulatory actions in ATLs.

## Introduction

Many learners in primary schools use Adaptive Learning Technologies (ALTs) in the Netherlands and around the globe (OECD iLibrary, 2016; Di Giacomo et al., 2016). These technologies allow learners to practice new mathematics, grammar and spelling skills on a tablet or Chromebook. ALTs are mostly used in blended classrooms, where alongside digital practice, teachers provide instruction and feedback (Molenaar and van Campen, 2016). Although ALTs have been found to improve learning (Aleven et al., 2016a; Faber et al., 2017), the question of how learners regulate their learning using these technologies remains largely unanswered. Great diversity in learners’ behavior during practice has been found with respect to the number of problems solved as well as the accuracy of problem-solving (Molenaar et al., 2019a). In addition to differences in prior knowledge, this variation could originate from differences in how learners regulate their learning (Arroyo et al., 2007; Paans et al., 2019). Yet little is known about how learners regulate their learning in ALTs (Winne and Baker, 2013; Bannert et al., 2017).

Although trace data from ALTs provide detailed insights into practicing behavior which can be used to detect how learners learn over time (Greller and Drachsler, 2012; Gašević et al., 2015), their intentions cannot be deduced from the data trace. A way to examine learners’ intentional regulation is to ask them to fill in feed-up and feed-forward reports (Hattie and Timperley, 2007), in which they set goals before a lesson and self-evaluate goal attainment after a practice session. This externally triggered goal setting and self-evaluation may influence learners’ practice behavior and learning outcomes. This study had two goals therefore: (i) to examine the effects of feed-up and feed-forward reports on regulation of practice behavior and learning outcomes; and (ii) to investigate how learners set goals and self-evaluate goal attainment and how this could be associated with their practice behavior and performance.

This exploratory study contributes to the objectives of the special issue as it deepens our understanding of how regulation and learning interrelate and co-evolve in digital environments. Methodologically we combined the advantage of elaborate data traces to understand practice behavior with insights into learners’ intentions measured by students’ self-reports. First, we elaborate on how learners regulated learning while practicing in ALTs. Second, we discuss how feed-up and -forward reports provided insight into learners’ intentions with regard to regulation and how it could affect learning as an external trigger for self-regulated learning.

### Regulation and Affective States in ALTs

Adaptive Learning Technologies are widely used to practice arithmetic, spelling and grammar in primary education in the Netherlands (Molenaar et al., 2016; Faber et al., 2017) and around the world (Aleven et al., 2016b). These technologies are often integrated in blended learning contexts. Teachers provide instruction in new knowledge or skills, after which learners continue to practice on their own devices while teachers give individual learners feedback. There are three main advantages of ALTs over paper-based practice: (i) ALTs provide learners with direct feedback on answers given (Faber et al., 2017); (ii) ALTs adjust problems to the needs of learners by estimating their current knowledge and/or the probability that they will solve the problem correctly (Corbett and Anderson, 1995; Klinkenberg et al., 2011); and (iii) ALTs provide teachers with concurrent feedback about learners’ performance in dashboards (Molenaar and Knoop-van Campen, 2018). Even though positive effects of ALTs on students’ learning have been found compared to traditional learning environments (Aleven et al., 2016b; Molenaar and van Campen, 2016; Faber et al., 2017), few studies have addressed how students regulate practice behavior in ALTs and how this affects their learning.

In order to understand how learners regulate their learning in an ALT, we drew on the COPES model of self-regulated learning (Winne and Hadwin, 1998). This theory defines learning as a goal-oriented process in which learners make conscious choices working toward learning goals (Zimmerman, 2000; Winne and Hadwin, 2017). In order to reach these goals, learners engage in cognitive activities (read, practice, elaborate) to learn a new topic. Metacognitive activities (orientation, planning, monitoring, and evaluation) help learners to control and monitor their learning to ensure effective and efficient learning (Veenman, 2013; Molenaar et al., 2019a). Affective states motivate learners to put in an appropriate level of effort to progress toward their learning goals (Azevedo et al., 2008). In the COPES model (Winne and Hadwin, 1998; Winne, 2018) regulation unfolds in four loosely coupled phases: (i) the task definition phase in which learners generate an understanding of the task; (ii) the goal setting phase in which learners set their goals and plan their actions; (iii) the enactment phase in which learners execute their plans working toward their goals; and (iv) finally the adaption phase which is activated when progress toward the goals is not proceeding as planned and adjustments in strategies, actions or tactics are required. These phases occur in the context of task conditions and operations performed by learners that lead to new knowledge and skills.

At the same time, we know that learners often fail to regulate their learning effectively (Azevedo et al., 2008). It is well established that learners often face a utilization deficiency (Winne and Hadwin, 2013), which is the failure to adequately activate the monitor and control loop during learning. This loop is at the heart of the COPES model and is largely dependent on goals learners set. Only after learners have set goals, can they evaluate their performance and diagnose progress (Winne and Hadwin, 2017). Research has indicated that, even for students in higher education, it is difficult to set goals in a way that drives monitoring and control (McCardle et al., 2017). In the enactment phase, learners compare performance and goals in cognitive evaluation to determine the need for adaptations. Without objective performance information, students are dependent on their own self-evaluation (Panadero et al., 2018). Up till now few studies have investigated self-evaluation of goal attainment in real learning sessions (Nederhand, 2018). During practicing, the calibration between self-evaluation of goal attainment and goals set is important for signaling self-regulatory actions. These actions take the form of “If, Then Else” sequences (Winne, 2010). For example, *if* a learner judges their goal to be reached, *then* practice activities can cease, *else* practice is continued. *If* progress is lower than expected, *then* the student must increase effort to increase learning, *else* keep effort at the same level. Therefore, in order to understand students intentions with regard to regulation, we focused on the signals learners deduce and examined how self-evaluation of goal attainment related to learners’ goals.

In this study, we examined calibration of goal attainment to understand the signals students deduce for regulation, whereas in most studies calibration refers to the alignment of a metacognitive judgment and a standard, most often test performance (Pieschl, 2009; Winne and Muis, 2011; Koriat, 2012). Most research investigates the “accuracy” of learners’ judgments compared to real performance (Pieschl, 2009). Although, as pointed out above, calibration of performance, i.e., how self-evaluation of goal attainment is related to actual performance, is not the signal that drives regulatory actions, it is important. It is a way to determine the validity of the signal students deduce from the calibration of goal attainment and the direction of possible inaccuracies. For example, *if* a student overestimates goal attainment, the signal used for regulatory actions indicates to stop practicing *and* calibration of performance indicates that the student has overestimated actual performance, *then* the student should continue to practice. In this case, the signal used for regulation is invalid and drives unwarranted adaptations. Especially when calibration of goal attainment and performance are biased in the same direction, over- or under-practicing is likely to occur. Although ample research has shown that young learners tend to overestimate their performance (Roebers, 2017), little is known about the standards they use to evaluate their performance nor about their ability to correctly self-evaluate goal attainment.

In addition to the emphasis on cognitive evaluation, the COPES model stresses that regulation is dependent on context (Winne and Hadwin, 2013). In ALTs learners mainly work on problems to further develop knowledge and skills on specific topics. Goal setting may help learners to evaluate progress and determine when to stop practicing or detect the need for adjustment. The ALTs’ adaptivity also supports learners’ regulation. Selected problems are adjusted to the student’s current performance, so that the technology partially overtakes monitoring and control from learners (Molenaar et al., 2019b). Nevertheless an important element of regulation remains the task of the students, namely adjustment of effort to maintain accuracy (Winne, 2010; Hadwin, 2011). Accuracy is viewed as a function of knowledge and effort and learners can regulate accuracy by adjusting one or both of these elements (Molenaar et al., 2019a). ALTs often provide direct feedback indicating whether an answer is correct. Learners can use this feedback to estimate their accuracy over the whole practice session. For example, learners making many mistakes should increase their effort. As such ALTs’ direct feedback has a function as a signal for adaptations. Direct feedback during practice provides explicit information to support regulation, but only when learners are able to process this information and translate it into meaningful adaptations. Even though some aspects of regulation are overtaken by the ALT, effort regulation remains the task of the student, which means that learners continue to control an important element of self-regulated learning.

To summarize, the control and monitoring loop in the COPES model explains how learners’ internal feedback functions and drives how they regulate accuracy and effort during learning. When learners are effectively regulating their learning, they set goals which they use to evaluate the need for adaptations. Calibration of goal attainment provides an important signal for regulatory actions and in ALTs direct feedback can function as a signal for adjustments during practice. Even though part of the regulation is overtaken by the ALT, young learners still need to regulate their accuracy and invest sufficient effort to ensure progress toward their goals. Great diversity in practice behavior raises the question of how capable learners are in regulating their practice behavior and what their intentions are in these contexts.

### How ALT Data and Interaction Influence Internal Regulation

Even though direct feedback available in an ALT may support cognitive evaluation, learners need to engage in this cognitive evaluation and translate the results into actions to actually impact their practice behavior. This is a complex process, especially for young learners. A failure to detect miscalibration will prevent the learner from making the right inferences and may lead to incorrect monitoring and trigger ineffective control actions. Further research into how learners set and self-evaluate goals, and how they interpret feedback as a signal, is essential to understand how learners regulate during learning in an ALT. Feed-up and feed-forward reports help learners set goals, self-evaluate goal attainment and explicitly formulate actions to improve their learning. These reports were originally developed as formative assessment tools and are known to support learning (Hattie and Timperley, 2007). Even though no explicit correlation with self-regulated learning has been found, recent discussions of SRL and formative assessment talk about the interaction between external and internal feedback (Panadero et al., 2018). A *feed-up report* is an external trigger to support learners to articulate *when* learning goals are reached (Hattie and Timberley, 2007). This helps them to set goals and standards for regulation. Standards help learners to set criteria that indicate *how* they can know when a learning goal has been reached. Consequently, feed-up reports are expected to support learners’ cognitive evaluations in the enactment phase of the COPES model. A *feed-forward* report is an external signal to trigger cognitive evaluation, i.e., to compare the goals set with self-evaluated goal attainment to evaluate progress. When learners establish a difference between their self-evaluated goal attainment and the standards, they realize that their progress is not as expected and adaptation is needed. This may signal re-evaluation of effort or a change in strategies. Feed-forward supports learners to explicitly state estimated performance and determine the need for control actions based on that.

In addition to feedback that signals the level of accuracy to learners, *feed-up* and *feed-forward* reports can be an external trigger to help them to effectively monitor and control their learning. Integrating direct feedback with feed-up and feed-forward during learning can support a comprehensive approach to stimulate regulation by supporting learners to set standards, i.e., learning goals (feed-up) and verbalize progress toward their learning goals (feed-forward). This in turn may drive adaptation, which could support young learners to optimize their regulation. Various techniques e.g., prompts (Bannert et al., 2009), scaffolding (Azevedo et al., 2008), and intelligent tutor systems (Azevedo et al., 2016) have been used previously to assist learners’ regulation in ALTs. Although these techniques are initially effective, they are less successful in sustaining regulation during learning in the absence of the tools. A drawback of these techniques is that they do not help learners to make explicit inferences about how their actions are related to progress toward their learning goals (Winne and Hadwin, 2013). The relation between performance, internal representations of the learning goals and goal attainment remains underspecified, making the contribution of practice to progress unclear.

## Purpose of the Study

Although ALTs support learning, the question of how learners regulate their practice behavior in ALTs remains unanswered. Diversity in the number of problems and the accuracy achieved among learners in this context requires more insight into students’ regulation. Trace data provide elaborate information about students’ practice behavior, but fail to provide insight into learners’ intentions. Goals learners set and their self-evaluation of goal attainment reflect those intentions and provide an important signal for regulatory actions. In addition, direct feedback may have a regulatory impact if learners have clear standards against which to evaluate their accuracy. In this study, we investigated how learners set goals and self-evaluate goal attainment using feed-up and feed-forward reports. These reports may function as external triggers to optimize learners’ internal feedback loop, which in turn affects their regulation and learning. Hence this study had two goals: (i) to examine how feed-up and feed-forward reports may support learners’ regulation and learning; and (ii) to investigate learners’ regulatory intentions and the signals they use as input for regulatory actions. We used an exploratory experimental pre-test post-test design executed as a field study in group 7 (students aged 10–11) arithmetic classes in Dutch primary schools.

The following research questions are addressed:

1. How do the feed-up and feed-forward reports affect learners’ effort, accuracy and learning?

Based on earlier research, we expected the feed-up report to trigger learners to articulate goals which could be used as standards in cognitive evaluation. The feed-forward report is intended to support learners to self-evaluate their goal attainment. This external trigger to regulate is expected to improve practice behavior (effort and accuracy) and learning. We expected that learners in the experimental condition would make more effort (hypothesis 1), be more accurate (hypothesis 2) and consequently learn more (hypothesis 3).

2. What signals do learners deduce during self-evaluation and how is self-evaluation of goal attainment related to actual performance?

Hardly any research has been done on self-evaluation of goal attainment, especially in young learners, and so we were unable to formulate any hypotheses. Previous research has indicated that young learners tend to overestimate their performance, which we also expected in this context.

3. What signals do learners pick up from direct feedback and how does self-evaluation of accuracy attainment relate to actual accuracy?

This is an exploratory question as the signaling role of direct feedback on self-evaluation of accuracy has not been previously studied. A higher calibration on accuracy attainment compared to calibration of goal attainment could indicate a signaling role of direct feedback.

4. How are students’ calibration values related to each other, to practice behavior (effort and accuracy) and to learning?

We explored how calibration of goal attainment and performance are related to further understand learners’ signals for regulatory action. We also explored whether and how calibration values are associated with practice behavior and learning.

## Materials and Methods

### Participants

The participants in this study were 71 group 7 learners. The three participating schools were located in the east of the Netherlands and had a diverse population. The learners were between 10 and 12 years old with a mean age of 11.17 years and 33 boys (46%) and 38 girls (54%) participated in this study. Classes were randomly assigned to the experimental condition [two classes (*n* = 37)] and the control condition [two classes (*n* = 34)]. Learners had to participate in at least 3 out of 4 lessons. Based on that criterion, three learners were excluded from the sample. Furthermore, five learners missed the pre-test and four learners did not participate in the post-test.

### Design

**FIGURE 1:**
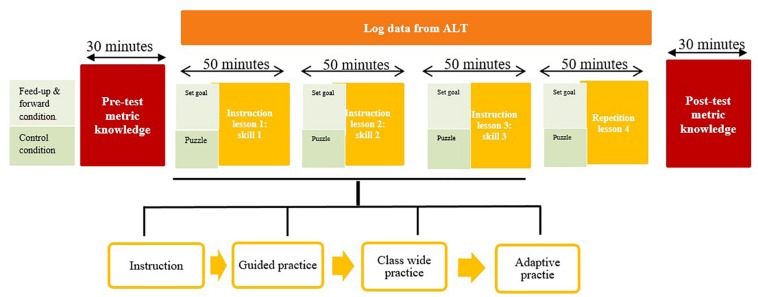
Study design.

This study was conducted with a quasi-experimental pre-test – post-test design (see Figure 1).

Learners in the experimental condition were asked to fill in the feed-up and feed-forward reports in every lesson. They set goals prior to every lesson and self-evaluated their progress at the end of the lesson. Learners in the control condition did a puzzle prior to every lesson to keep the total time investment equal over the two conditions. Learners received instruction and practiced the three arithmetic skills during three lessons of 55 min each on three consecutive days. The design of each lesson followed the direct instruction model including teacher instruction, guided practice, class-wide practice and individual practice. The pre-test took place prior to the first lesson and learners did the post-test after completion of all the lessons. In the fourth lesson learners were instructed to practice the skills which they needed most practice in.

### Materials

#### Feed-Up and Feed-Forward Reports

**FIGURE 2:**
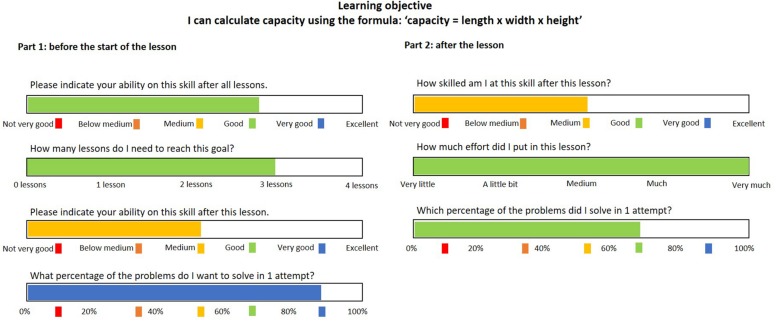
The feed-up (part 1) and feed-forward (part 2) reports.

In the *feed-up* report, learners formulated their learning goals and standards to evaluate performance and progress. At the start of each lesson (first three lessons), learners in the experimental group were asked to answer four questions regarding their learning goals: (1) How skilled do you want to become at this particular subskill? (2) How many lessons do you need to reach that goal? (3) How skilled do you want to become in this particular lesson? (goal for performance) (4) What percentage of problems will you solve correctly at the first attempt? (goal for accuracy). Learners answered all questions by sliding the bars below the questions (see the left side of Figure 2). The sliders represented a percentage the learners reached between 0 and 100. The chosen colors represented different ability levels which were also used in the teacher report on the ALT and learners were familiar with the color coding.

In the *feed-forward* report, learners were asked to self-evaluate goal attainment and progress toward their learning goal. After each lesson (first three lessons), they were asked to answer three questions: (1) What is your current ability level on the subskill studied today? (self-evaluation of goal attainment) (2) How much effort did you put into today’s lesson? (3) What percentage of problems did you solve correctly in one attempt? (self-evaluation of accuracy attainment). Learners answered by sliding the bars below the questions (see the left side of Figure 2). Next, learners were asked to compare part 1 with part 2 to determine their *progress* and to see how far they were from reaching their goal. They were asked to indicate how often they received help from the teacher, whether they tried harder to solve a difficult problem, and whether they consulted hints in the ALT to solve the problem. They also had to indicate how satisfied they were with their learning during the lesson and what they would improve in the next lesson.

Before the fourth lesson (the rehearsal lesson), the learners were asked to indicate their ability on all three subskills, set goals for this last lesson and determine which subskill(s) they needed to work on in the rehearsal lesson. Again, learners indicated their ability scores at the end of the rehearsal lesson, evaluated their progress at the end of the lesson, explicitly indicated whether or not they had reached their goal, and explained their progress. This method meant that the feed-up, feed-forward cycle was repeated four times during the experiment.

#### The Adaptive Learning Technology

The ALT used in this study is widely used for spelling and arithmetic education throughout the Netherlands. This technology is applied in blended classrooms in which the teacher gives instruction after which learners practice on their tablets. First, learners practiced in the class-wide practice stage on non-adaptive problems which were the same for each student in the class. Next, learners worked on adaptive problems, which were selected after each problem solved based on an estimate of the learner’s knowledge called the ability score (Klinkenberg et al., 2011). This score was calculated by a derivative of the ELO algorithm (Elo, 1978). Based on the learner’s ability score, the ALT selected problems with a probability of 75% that the learner would answer the problem correctly. After a learner had answered approximately 25 problems, the system had a reliable indicator: the ability score. This ability score was used as an indicator of performance. The difference between the previous ability score and the new score was used as an indicator of progress. Learners were also given direct feedback (correct or incorrect) after entering an answer to a problem and teachers could follow learners on teacher dashboards (Molenaar and Knoop-van Campen, 2018).

#### Subskills Learned

**FIGURE 3:**
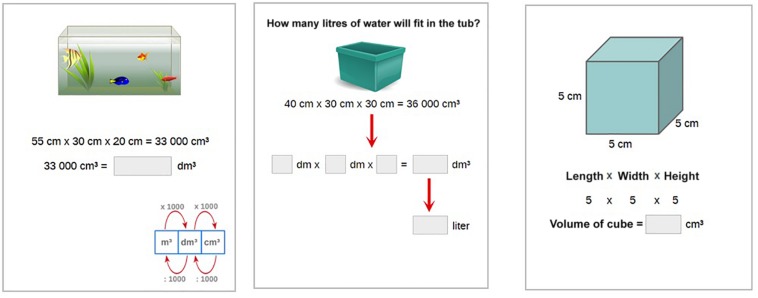
Examples of items for the three subskills in the pre- and post-test.

The three subskills all included different aspects of measurement of capacity (see Table 1). The Dutch metric system units for measuring capacity were used. The problems related to the first subskill “Calculate capacity using the formula: “capacity = length × width × height” were relatively easy because learners were given a formula to solve the problem. Examples were also used in this subskill to support learners” problem-solving. The problems related to the second subskill “Convert from common capacity units to cubic meters” (cm^3^, dm^3^, m^3^) were of medium difficulty. Finally, problems within the third subskill “Convert cubic meter units (cm^3^, dm^3^, m^3^) to liter units” (cl^3^, dl^3^, l^3^) were hard, as learners were asked to do the conversion without a formula (see Figure 3 for more examples).

**TABLE 1:** Examples of problems for each subskill.

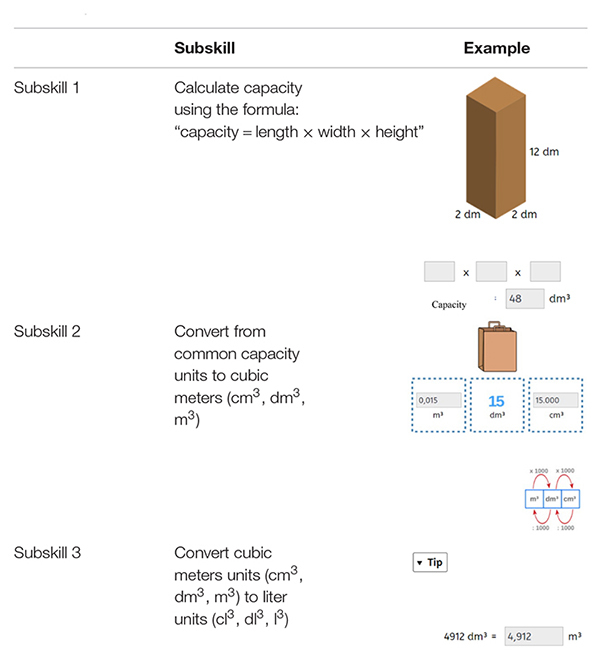

### Measurements

#### Pre- and Post-test

The pre- and post-test consisted of 24 items, 8 items per subskill. The items in the pre- and post-test were structurally similar but different numbers were used. The difficulty level of the items, as indicated by the ALT, was used to balance both tests. Figure 3 provides examples of the items for each subskill. The overall Cronbach’s alpha for the whole pre-test was 0.93, with 0.94 for subskill 1, 0.93 for subskill 2, and 0.74 for subskill 3, respectively. The overall Cronbach’s alpha for the post-test was 0.91, with 0.74 for subskill 1, 0.92 for subskill 2, and 0.78 for subskill 3, respectively. Learning gain was calculated as the difference between pre- and post-test. The values (given in the results section below) indicated that there was limited evidence for a ceiling effect, requiring a more complex measure of learning gain.

#### Measures From the ALT

The knowledge a student acquired on a subskill was expressed in the ability level as calculated by the ELO algorithm. This score was given as a number between 0 and 600. In order to compare this value with the students’ goals, we translated the ability score into a percentage. The logs of the ALT stored data of the learners’ practice activities, including a date and time stamp, student identifier, problem identifier, learning objective identifier, ability score after each problem and accuracy of the answer given. Based on this information indicators of effort and accuracy were calculated. Effort was measured by one indicator per subskill: the number of unique problems a student completed to practice this subskill. Accuracy was calculated by dividing the number of correctly answered problems by the total number of problems completed. Table 2 provides an overview of all measures calculated and their definition.

**TABLE 2:** Overview of learning, effort, and accuracy measures.

**Learning measures**	**Definition**
Prior knowledge	Pre-test, one per subskill
Post-knowledge	Post-test, one per subskill
Gain	Post-test/pre-test per subskill

**Process measures**	**Log file data**

Unique problems	Number of unique problems completed per subskill
Accuracy unique problems	Correct unique problems/total unique problems completed

#### Measures Taken From the Feed-Up and Feed-Forward Reports

A number of measures were taken from the feed-up and feed-forward reports used in the first three lessons (see Table 3 for an overview). The feed-up report was used to measure the overall learning goal set per subskill, the lesson goal for each lesson and the goal for accuracy. The feed-forward report measured *self-evaluation of goal attainment* and *self-evaluation of accuracy attainment*. All these values were measured on a scale from 0 to 100%. Self-reported effort was measured on a scale from 1 to 5. The calibration values were calculated based on the values in the feed-up and feed-forward reports. *Calibration of goal attainment* was calculated by deducting the goal set from the self-evaluation of goal attainment. *Calibration of performance* (typically referred to as calibration accuracy) was calculated by deducting the accuracy goal from the self-evaluation of accuracy attainment. These two values can be seen as signals for regulatory actions. In order to assess the correctness of these signals, *calibration of performance* was calculated by deducting actual performance from the self-evaluation of goal attainment and *calibration of accuracy* was calculated by deducting actual accuracy from self-evaluation of accuracy attainment.

**TABLE 3:** Measures taken from the feed-up and feed-forward reports.

	**Description**	**Scale**
**Feed-up**		
Overall goal set	The ability level the student ultimately wants to achieve for this subskill	0–100%
Lesson goal set	The ability level the student wants to achieve for the subskill during the first lesson	0–100%
Accuracy goal set	The percentage of problems a student wants get right at the first attempt	0–100%
**Feed-forward**		
Self-evaluation of goal attainment	The performance in ability level a student perceives to have achieved on the subskill during the lesson	0–100%
Self-evaluation of accuracy attainment	The percentage of problems a student perceived to have got right at the first attempt	0–100%
Self-reported effort	How hard the student worked during the lesson	Scale between 1 and 5
**Calibration**		
Calibration of goal attainment	Self-evaluation of goal attainment – lesson goal set	
Calibration of accuracy attainment	Self-evaluation of accuracy attainment – accuracy goal set	
Calibration performance	Self-evaluation of goal attainment – actual performance	
Calibration of accuracy	Self-evaluation of accuracy attainment – actual accuracy	

### Procedure

On the first day learners took the pre-test (30 min) after which the first instruction lesson of 45 min was given. The two other instruction lessons and the repetition lesson were given on separate consecutive days following the first lesson. On the fifth day learners took the post-test (30 min). Each instruction lesson started with 10-min instruction given by the teacher. This was standardized by using an instruction protocol. Afterward, the teacher practiced six to eight problems with the learners in guided practice. Then the learners continued to work on problems within that particular subskill. First, they completed a set of non-adaptive problems (15 problems) which were the same for all learners in the class. They then worked on adaptive problems for the remaining time in the lesson. In the fourth lesson the three subskills of the previous lessons were repeated and practiced with adaptive problems. Learners were instructed to select subskills depending on their learning goals.

### Analysis

In order to assess how the feed-up and feed-forward intervention affected effort and accuracy, a MANOVA analysis was performed with effort on skill 1, skill 2, and skill 3 as within-subject factor and condition as between subject factor. A repeated measurement MANOVA was used to assess how the feed-up and forward intervention affected learning with the pre- and post-test scores (time) on skill 1, skill 2, and skill 3 (skill) as within-subject factor and condition as between subject factor.

This analysis consisted of three steps: (i) we addressed learners’ *intentions regarding regulation*; (ii) we assessed the *signals learners deduced*; and (iii) we determined the *correctness of the signals*.

In order to understand learners’ *intentions regarding regulation*, the goals they set, self-evaluation of goal attainment, accuracy goals set and self-evaluation of accuracy attainment from the students’ feed-up report were reported. Next, to investigate the *signals learners’ deduced* during cognitive evaluation, we analyzed the calibration of goal-attainment (self-evaluation of goal attainment – goals set). We calculated an absolute difference to understand the distance between the goals set and the learners’ estimation of performance after the lesson. The relative difference was used to understand the bias learners have in their signals. Bias may be overestimation when learners assess their goal attainment to be higher than their goals set or underestimation when they assess goal attainment to be lower than their goals set. Finally, to determine *the correctness of the signals* the calibration of performance was calculated (self-evaluation of goal attainment – actual performance). Again, the absolute and relative values were reported. We speak of overestimation when learners’ self-evaluation of goal attainment was higher than actual performance and underestimation when it was lower than their goals set. The same logic in three steps was followed for calibration of accuracy and goal attainment. Finally, correlations were calculated in order to understand the relations between the calibration values.

## Results

Table 4 shows the descriptive statistics of the pre-test, post-test, gain, effort in the number of unique problems solved and accuracy while solving these problems.

**TABLE 4:** Descriptive statistics.

	**Subskill 1**				**Subskill 2**				**Subskill 3**			
			
	**Con**		**Exp**		**Con**		**Exp**		**Con**		**Exp**	
						
	***M***	***SD***	***M***	***SD***	***M***	***SD***	***M***	***SD***	***M***	***SD***	***M***	***SD***
Pre-test	7.36	1.45	6.92	1.99	5.58	2.83	2.69	2.81	3.55	2.17	2.33	1.84
Post-test	7.73	0.52	7.73	1.46	7.36	0.99	6.72	2.09	4.97	2.16	4.47	1.65
Gain	0.36	1.54	0.11	1.85	1.91	2.72	4.03	2.79	1.44	1.85	2.14	2.10
Effort	39.12	6.69	35.58	8.85	42.50	13.18	41.56	13.55	44.09	9.74	44.33	18.66
Accuracy	0.92	0.07	0.87	0.13	0.85	0.13	0.71	0.20	0.73	0.13	0.63	0.14

### Effect of Feed-Up and Feed-Forward Reports on Effort and Accuracy

For effort there was a significant main effect on skill *F*(2, 67) = 5.41, *p* < 0.01 indicating that learners showed different effort on the three subskills. There was no significant interaction between skill and condition *F*(2, 67) = 0.41, *p* > 0.05: in the experimental condition learners did not show more effort compared to learners in the control condition (hypothesis 1, rejected).

For accuracy there was a significant main effect on skill *F*(2, 67) = 76.91, *p* < 0.01 indicating that learners showed different accuracy on the three subskills. There was significant interaction between skill and condition *F*(2, 67) = 0.3.13, *p* < 0.05: in the experimental condition learners showed lower accuracy compared to learners in the control condition (hypothesis 2, rejected).

### Effect of Feed-Up and Feed-Forward Reports on Learning Outcomes

**FIGURE 4:**
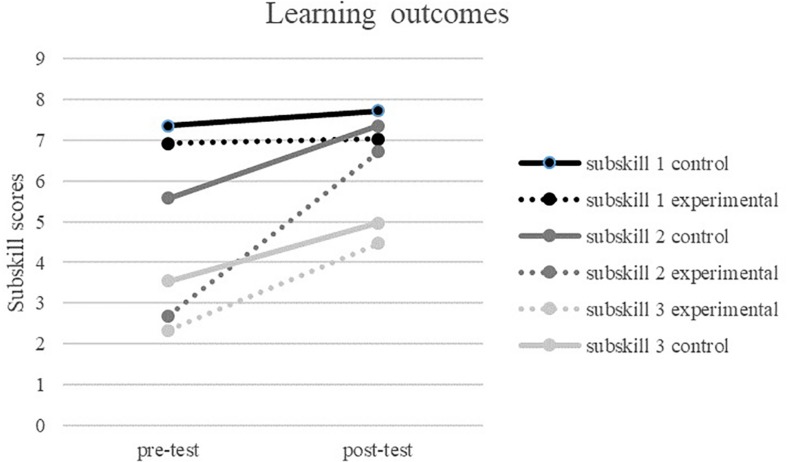
Learning over the lessons per subskill indicating pre-test and post-test.

There was a significant main effect of time *F*(1, 67) = 109.45, *p* < 0.001: learners scored higher on the post-test compared to the pre-test. There also was a main effect of condition *F*(1, 67) = 1507.68, *p* < 0.001: learners in the experimental group scored differently from learners in the control group; and a main effect of skill *F*(2,67) = 404.89, *p* < 0.001, learners scored differently on the three skills. In addition, there was an interaction effect between skill and condition, *F*(2, 67) = 13.27, *p* < 0.025, skill and time *F*(2,67) = 61.71, *p* < 0.001 and time and condition *F*(1,67) = 20.95, *p* < 0.01. Finally, there was a three-way interaction between skill, time and condition *F*(2, 67) = 13.59, *p* < 0.01. Follow-up analysis revealed that the experimental group scored lower on pre-test for subskills 2 and 3 compared to the control condition, whereas there were no differences at pre-test on subskill 1 (see Figure 4). The experimental group scored lower on post-test on subskill 1 compared to the control group, whereas for subskills 2 and 3 no significant differences were found. Finally, the experimental group showed a stronger growth over time for subskill 2 compared to the control group, whereas no differences in growth were found on subskills 1 and 3 (hypothesis 3, partially supported).

### Relations Between Learners’ Self-Evaluation of Goal Attainment and Actual Performance

*The learners’ intentions regarding regulation* were entered in the feed-forward report. The lesson goals set by the students, their self-evaluations of goal attainment and their actual performance data are shown in Table 5. Before the lesson, the average goal set for a lesson was between 71% for lesson 1 and 76% for lesson 3. After the lesson, learners’ self-evaluation of goal attainment was 76% on average which remained similar over the three lessons. Ability level, indicating actual performance, was available for 33 learners after lesson 1 but only for 6 learners after lesson 2 and 22 learners after lesson 3. The remaining 29 learners on skill 2 and 14 learners on skill 3 did not solve enough problems to calculate an accurate ability score. For skill 1 the average ability score was 80%, for skill 2 the average was 87% (6 learners) and for skill 3 the ability score was 83%.

**TABLE 5:** Learners’ *intentions regarding regulation*: lesson goals, self-evaluation of goal attainment, and actual performance after the lesson.

		**Lesson goals**		**Self-evaluation**		**Actual**	
		**set**		**of goal attainment**		** performance**	
				
	***n***	***M***	**SD**	***M***	**SD**	***M***	**SD**
Lesson 1	35	71.71	19.01	75.14	19.15	80.24^1^	6.43
Lesson 2	36	73.16	16.42	77.36	17.01	87.67^2^	7.35
Lesson 3	36	76.95	16.57	76.67	20.14	83.86^3^	5.23

*^1^*n* = 33, ^2^*n* = 6, ^3^*n* = 23.*

*The signals learners deduced* to drive regulation in cognitive evaluation varied between self-evaluation of goal attainment and goals set which is called calibration of goal attainment. The average absolute goal attainment showed a 13% difference between learners’ self-evaluation of goal attainment and goals set (see Table 6). This number was similar over the three lessons and indicates that learners on average were incorrect by 13%. With respect to bias in the goal attainment calibration, for lessons 1 and 2 the average relative calibration was positive. This indicates a trend toward overestimation of goal attainment, i.e., the self-evaluated goal attainment was higher than goals set. For lesson 3, this relative goal attainment value was approaching 0, indicating a trend toward calibration between self-evaluated goal attainment and goals set. When we further analyzed the bias in learners’ goal attainment calibration, we found that the number of learners that perfectly calibrated increased over time from 11 in lesson 1–17 in lesson 3. The number of learners that overestimated their goal attainment remained similar at around 9 learners and the number that underestimated goal attainment reduced over time from 14 in lesson 1–9 in lesson 3.

**TABLE 6:** *Signals for regulation*: overview of absolute and relative calibration of goal attainment and performance per lesson.

		**Absolute calibration**		**Relative calibration**			**Relative calibration**		**Relative calibration**	
		**goal attainment**		**goal attainment**			**performance**		** performance**	
						
	***n***	***M***	**SD**	***M***	**SD**	***n***	***M***	**SD**	***M***	**SD**
Lesson 1	34	15.29	13.76	2.94	20.52	33	12.83	10.75	−4.47	16.27
Lesson 2	36	12.92	13.00	3.75	18.06	6	9.89	4.89	−2.67	11.52
Lesson 3	36	11.94	12.83	–0.28	17.65	22	14.69	15.61	−5.71	20.87

*Correctness of the signal* was evaluated by calibration of performance. Overestimation of goal attainment can function as a regulatory signal to stop practicing, as according to the self-evaluation the goal has been achieved. This may be an erroneous signal when the self-evaluation and the actual performance are not aligned. For this reason, we examined calibration of performance, the relation between self-evaluated goal attainment and actual performance. This showed that on average learners were 13% inaccurate in their estimations. With respect to bias, the average relative goal attainment calibration was negative for all three lessons. This indicates a trend toward under-estimation (the self-evaluated goal attainment was lower than the actual ability level). On average learners estimated their performance lower than their actual ability level. When we further analyzed the bias in learners’ performance calibration, the majority of the learners, approximately 60%, underestimated their performance in lessons 1 and 3 and a smaller group, around 40%, overestimated their performance (see Table A1 in the Appendix).

The combination of overestimation of goal attainment and performance is especially problematic as errors in both calibrations reinforce an unwarranted reduction in effort. Similarly, underestimation of goal attainment combined with an underestimation of performance underlie an unnecessary increase in effort. There was a significant positive correlation (*r*_skill__1_) = 0.60, *p* < 0.01 and (*r*_skill__3_) = 0.75, *p* < 0.01 between calibration of goal attainment and performance for lessons 1 and 3. This indicates that when learners over or underestimate goal attainment, they also tend to over or underestimate performance. This points toward a reinforcing effect that may induce an erroneous regulation of effort.

### Relations Between Learners’ Self-Evaluation of Accuracy and Actual Accuracy

*The intentions of learners with regard to regulation* of accuracy were also entered in the feed-forward report. Accuracy can function as a signal for learners to better understand their performance. Table 7 provides the descriptive data on accuracy of goals set before the lesson, self-evaluation of accuracy after the lesson and actual accuracy. Before the lesson, the average of goal set for accuracy was 75%, which was similar for all three lessons. After the lesson, learners’ self-evaluation of accuracy reduced over the three lessons from 84% in lesson 1–74% in lesson 3; this reduction was not significant *F* (32, 2) = 2.88, *p* = 0.07. This was in line with a significant reduction in actual accuracy *F*(32, 2) = 42.82, *p* < 0.001) over the lessons from 81% in lesson 1–73% in lesson 3.

**TABLE 7:** Overview of the *intentions of learners for regulation*: accuracy of goals, self-evaluated accuracy, and actual accuracy per lesson.

		**Accuracy of goals**		**Self-evaluated**			
		**set before**		**accuracy after**		**Actual**	
		**the lesson**		**the lesson**		**accuracy**	
				
	***n***	***M***	**SD**	***M***	**SD**	***M***	**SD**
Lesson 1	35	72.63	16.10	84.09	16.77	91.02	11.61
Lesson 2	36	77.63	17.01	80.39	18.88	82.77	19.47
Lesson 3	36	76.31	16.57	74.72	14.63	73.23	14.62

*The signals learners deduced for regulation of accuracy*: During practice learners received direct feedback which can be a signal for them to better understand their level of accuracy. The relation between accuracy of goal and self-evaluated accuracy is called the accuracy attainment calibration. Again, there is an absolute and a relative value. The average absolute accuracy attainment calibration was 13%, which indicates a difference between the accuracy set and self-evaluated accuracy (see Table 8). This number was similar over the three lessons. With respect to bias in the accuracy attainment calibration, in lessons 1 and 2 the average relative calibration was positive, indicating a trend toward overestimation of accuracy attainment. For lesson 3 this value was negative, indicating a trend toward underestimation of accuracy attainment. When we further analyzed the bias in learners’ self-evaluation of accuracy, it appeared that the group of learners that perfectly calibrated increased over time from 8 in lesson 1–15 in lesson 3. This indicates that learners’ estimates of their current level of accuracy were similar to their accuracy goals. The number of learners that underestimated their accuracy attainment reduced over the three lessons from 22 in lesson 1–8 in lesson 3. The number of learners that overestimated their accuracy attainment increased from 4 in lesson 1–13 lesson 3. Hence, the absolute calibration of accuracy attainment was comparable to the absolute calibration of goal attainment, but the relative calibration was somewhat higher for accuracy attainment. This shows little difference between the two calibration values which does not make the case for a signaling role of accuracy.

**TABLE 8:** *Correctness of the signals*: overview of absolute and relative calibration of accuracy attainment and accuracy per lesson.

		**Absolute calibration**		**Relative calibration**		**Absolute calibration**		**Relative calibration**	
		**accuracy attainment**		**accuracy attainment**		**of accuracy**		**of accuracy**	
					
	***n***	***M***	**SD**	***M***	**SD**	***M***	**SD**	***M***	**SD**
Lesson 1	35	14.15	12.05	10.91	15.12	9.89	10.83	–4.74	13.96
Lesson 2	36	12.19	11.92	3.03	16.90	12.40	12.68	1.60	17.78
Lesson 3	36	12.36	15.04	–4.58	19.03	14.93	12.46	3.73	19.27

*Correctness of the signals*: Overestimation of accuracy attainment can function as a trigger to reduce effort as self-evaluation indicates the goal has been achieved. This may be an erroneous signal when the self-evaluation and the actual accuracy are not aligned. Calibration of accuracy shows that average absolute calibration of accuracy was 10% for lesson 1, increasing toward 15% for lesson 3 (see Table A2 in the Appendix). With respect to bias, in lesson 1 the relative accuracy was negative, indicating a trend toward underestimation. Yet, for lessons 2 and 3 the relative accuracy was positive, indicating a trend toward overestimation. When we further analyzed the bias in learners’ accuracy calibration, we found that about half of the learners underestimated their accuracy and the other half overestimated their accuracy in all three lessons.

The combination of overestimation of accuracy attainment and accuracy may be especially detrimental for learners as errors in calibration reinforce an unwarranted regulation of effort. Similarly, underestimation of accuracy attainment combined with an underestimation of accuracy leads to an unnecessary increase in effort. There was a significant positive correlation (*r*_skill__1_) = 0.39, *p* < 0.05 and (*r*_skill__3_) = 0.35, *p* < 0.05 between calibration of accuracy attainment and accuracy for lessons 1 and 3. For lesson 2 the correlation was not significant (*r*_skill__1_) = 0.24, *p* > 0.05. This indicates that for skills 1 and 3, when learners under or overestimated accuracy attainment, they also tended to over or underestimate accuracy. This points toward a reinforcing effect that may induce an erroneous regulation of effort, but the association was lower than that between calibration of goal attainment and performance.

### Relation Between Calibration and Practice Behavior and Learning Outcomes

First, we assessed the relationship between the calibration values. We found a significant positive correlation between calibration of goal attainment and calibration of accuracy attainment for lesson 1 (*r*_skill__1_) = 0.40, *p* < 0.05 and 2 (*r*_skill__2_) = 0.40, *p* < 0.05. This indicates that learners’ bias in self-evaluation of performance and accuracy were linked. There was a significant positive correlation between calibration of accuracy and calibration performance for lesson 1 (*r*_skill__1_) = 0.60, *p* < 0.01. This indicates that self-evaluation, actual performance and accuracy were only related for the easy subskill but not for lesson 3.

Finally, we found a significant correlation between learners’ accuracy (the percentage of correctly answered problems) and calibration of accuracy for subskill 2 (*r*_skill__2_) = 0.35, *p* < 0.05. This indicates that learners who show high accuracy tend to estimate their accuracy more correctly. We found a significant correlation between learners’ effort (number of problems) and the calibration of accuracy, but only for skill 1 (*r*_skill__1_) = −0.43, *p* < 0.05. During lesson 1 when learners were more accurate, they solved more problems.

## Discussion

This study aimed to understand how learners regulate learning in ALTs. Next to trace data that provide insight into regulation of practice behavior, learners’ intentions for regulation were examined using feed-up and feed-forward reports. These reports acted as an external trigger to elicit goal setting and self-evaluation and were therefore expected to affect internal regulation. We hypothesized that feed-up and forward reports would have positive effects on regulation of practice behavior and learning. Subsequently learners’ intentions regarding regulation were further analyzed, examining how their evaluation of goal attainment functioned as a signal to drive regulatory actions. The correctness of this signal was assessed by examining the relation between self-evaluation and actual performance. We also examined the role of direct feedback as a signal for effort regulation. We investigated accuracy goals set to understand learners’ intentions. The calibration of accuracy attainment was used to understand the signal learners deduced to regulate accuracy during learning. In order to understand the correctness of this signal the relation with actual accuracy was investigated. Hence, self-evaluation and calibration were assessed to understand how learners engaged in cognitive evaluation and made decisions for adaptation to guide their practice behavior and learning.

We found no conclusive evidence that the feed-up and forward reports affected learning. We did find that learners in the experimental condition showed more growth of knowledge during the lessons than the control group. However, these learners also had less prior knowledge than the control group, which may have induced these results. Moreover, we did not find any differences between the experimental and the control group with respect to effort these learners put in. We did find a significant difference between the conditions on accuracy: the experimental condition showed lower accuracy than the control condition. Again, less prior knowledge may underlie these differences. Due to initial differences on prior knowledge between the conditions, it is difficult to draw definite conclusions about the effect of the feed-up and feed-forward reports.

To further understand learners’ intentions with regard to regulation, we investigated the goals learners set in the feed-up and feed-forward reports. The relation between these goals and learners’ estimates of performance were the signals learners deduced during cognitive evaluation. We found that learners were inaccurate in their self-evaluation of goal attainment. The relative calibration of goal attainment showed a positive bias for lessons 1 and 2, which indicated that learners tended to overestimate their goal attainment, producing a signal “*stop practicing, the goal has been reached.*” For lesson 3, we found a negative relative calibration of goal attainment which indicated that learners set higher goals than they obtained according to self-evaluated performance. This signal was “*continue to practice the goal has not yet been reached.*” Overall, we saw an increase in calibration over the lessons, which means that learners more often believed they had reached their goal during a lesson. Still a quarter of the learners underestimated and believed they should continue to practice. Half of the learners for lesson 1 to one third of the learners for lesson 3 overestimated and believed they had reached their goal.

In order to understand the correctness of the signals, we continued to look at the relation between self-evaluation of goal attainment and actual performance. We could only perform this analysis for lessons 1 and 3, because for lesson 2 the ALT could only calculate a valid ability score for six learners. The calibration of performance showed that learners on average were inaccurate. The relative performance calibration was negative for all lessons, indicating a tendency for learners to underestimate their performance. Deeper exploration of the calibration values showed that in all three lessons approximately half of the learners underestimated and the other half overestimated their performance. This is surprising as most research indicates that young learners tend to overestimate their performance (van Loon et al., 2013; Roebers, 2017). This may indicate that the feed-up and feed-forward intervention did affect our learners’ cognitive evaluation.

Calibration of goal attainment and performance were compared to understand how correct were the signals learners deduced. We found high positive correlations for lessons 1 and 3. Thus calibration of goal attainment and performance were highly related. When learners overestimated their goal attainment, which was the case for one third to half of the learners, they were also very likely to overestimate their performance. When translating this into “if then else” sequences, the signal “*stop practicing*” was most likely to occur when goals had not actually been reached. This error in the regulatory signal may have led to under-practicing. In a similar vein, when learners underestimated their goal attainment, which was one quarter of the learners, their actual performance was likely to be higher. In these cases, the signal “*continue to practice*” occurred when learners had in fact reached their goal leading to over-practicing. Hence, learners were likely to deduce inaccurate signals that drove their cognitive evaluation during the execution phase of the COPES model. This meant that learners were unable to accurately monitor their learning and consequently were likely to initiate incorrect control actions. Performance feedback could help learners to evaluate their progress more accurately and deduce valid signals to drive regulatory action (Panadero et al., 2018). Previous research has indicated that self-evaluation in feed-up and feed-forward reports supports learning, other studies have emphasized the need for performance feedback to actually affect regulation (Foster et al., 2017). The rationale is that in order to engage in cognitive evaluations learners need reliable, revealing, and relevant data in order to be able to draw valid inferences about their own learning process (Winne, 2010). Although the young learners in this study showed less inclination to overestimate compared to earlier research, the analysis above suggests that goal setting and cognitive evaluation alone were not enough to ensure learners deduced effective signals to drive regulation.

The role of direct feedback on learners’ ability to assess accuracy during practice was examined to see if this would help them to deduce more accurate signals during learning. The average absolute calibration of accuracy attainment was inaccurate. The average relative calibration values were positive for lessons 1 and 2, indicating overestimation and signaling to learners to reduce effort, and negative for lesson 3, demonstrating underestimation eliciting increased effort. There was an increase in calibration over the lessons, which meant that learners more often indicated that they had reached accuracy goals during practice. This increase in calibration caused a decrease in underestimation from over half of the learners in lesson 1 to about one fifth in lesson 3. Overestimation went up from one tenth in lesson 1 to one third of the learners in lesson 3.

The relation with actual accuracy helped us understand the correctness of this signal. The average absolute calibration of accuracy was again 13%. The relative calibration of accuracy was negative for lesson 1, where learners underestimated their accuracy and positive for lessons 2 and 3, where learners overestimated their accuracy. Further analyses indicated that calibration was low and reduced over the lessons. Underestimation reduced over the lessons from half to one third of the learners and overestimation increased from one third to two thirds of the learners. This was in line with the increase in difficulty of the skills over the lessons. Once again, the results indicate that learners were unable to accurately monitor their effort, deduced wrong signals that, when translated into control actions during the execution phase, would not support effective regulation.

Next, we compared the calibration of accuracy attainment and accuracy to understand the correctness of the signals learners deduced. We found medium positive correlations for lessons 1 and 2, but not for lesson 3. Again, the signals learners deduced were directed in the same direction. Overestimation in accuracy attainment was related to overestimation of accuracy. Half of the learners deduced the signal to reduce effort when they should have increased effort and similarly the other half of the learners inferred that they should increase effort when they should have reduced effort. This again provides evidence of inaccurate use of signals even though learners had received explicit direct feedback during practice. The problem may lie in the fact that direct feedback provided information on the local level, i.e., per problem (Pieschl, 2009), whereas accuracy judgments were made on the global level, i.e., over a number of problems. It may be that young learners find it hard to translate information from the local to the global level. Yet the association between calibration values was less strong for accuracy than for performance. Based on this finding we speculate that direct feedback may indeed have helped the learners to evaluate their accuracy more effectively than their performance.

Finally, we addressed how calibration values were related to each other and we found that calibration of goal attainment and accuracy attainment were associated for lessons 1 and 2. This indicates that the signal learners deduced based on self-evaluation were related to each other. For calibration of performance and accuracy, we only found a relation for lesson 1. This indicated that the bias between self-evaluated and actual performance and accuracy only existed for the easier subskill. Calibration values and practice behavior showed no association with respect to the number of problems solved nor with learning. We did find that accuracy and calibration of accuracy were related, indicating that learners with high accuracy tended to estimate their accuracy more correctly.

Limitations of this study were the fact that prior knowledge was different between the control and the experimental condition at the start of the experiment and so effects of the feed-up and feed-forward intervention could not be determined exclusively based on the experiment. However, the results of the in-depth analysis of learners’ cognitive evaluations did provide us with clear evidence that feed-up and feed-forward reports without performance feedback did not support young learners to deduce correct signals for regulatory actions. Moreover, although the sample size of the experimental group was sufficient to obtain more insights into learners’ regulation intentions, it was not large enough to engage in follow-up analysis of clusters of over and underestimating learners. Finally, for most students the actual performance (ability score) could not be determined by the ALT at the end of lesson 2 and, for lesson 3, there may have been a bias in the 22 students that did receive a score at the end of the lesson compared to the 13 students that did not receive an ability score. This research clearly emphasized the need for performance feedback during feed-forward interventions to increase the correctness of the regulatory signals that students deduce. Even direct feedback after each problem did not help students to correctly estimate their accuracy level during a lesson. Future research should investigate how learners benefit from performance feedback in a feed-forward report and if that influences practice behavior and learning. In addition to performance feedback, explicit information on their global accuracy level could be made available to students to support their regulation. Moreover, it would be interesting to assess in future studies how learners’ intentions, the signals they deduce and correctness of those signals changes over time.

## Conclusion

Although research has found evidence for positive effects of ALTs on learning, it has also found that the signals learners deduce to drive regulatory actions are mostly incorrect. We found no conclusive effects of the feed-up and feed-forward reports on learners’ practice behavior and learning. Furthermore, we found that young learners’ self-evaluations of goal attainment and performance were biased. Contrary to other research, we found that learners both over- and underestimated performance which was strongly associated with the over- or underestimation of goal attainment. Hence the signals learners used to drive regulation were often incorrect, which was likely to have induced over- or under-practicing. Similarly, we found a bias in self-evaluation of accuracy and accuracy attainment. Learners again over- or underestimated accuracy, which was associated with over- or underestimation of accuracy attainment, which may in turn have affected effort regulation. Yet the relation was less strong compared to performance, indicating that learners were supported by direct feedback in their accuracy judgment. We concluded that goal setting and self-evaluation in feed-up and feed-forward reports is not enough to deduce valid regulatory signals. Our results emphasize that young learners deduced inaccurate signals to drive their regulation and therefore needed performance feedback to support correct self-evaluation and to correctly drive regulatory actions in ALTs. This exploratory study has deepened our understanding of how regulation and learning interrelate and co-evolve in digital environments.

## Data Availability Statement

The datasets generated for this study are available on request to the corresponding author.

## Ethics Statement

The studies involving human participants were reviewed and approved by the Ethics Committee Faculty of Social Sciences. Written informed consent from the participants’ legal guardian/next of kin was not required to participate in this study in accordance with the national legislation and the institutional requirements.

## Author Contributions

IM wrote the sections “Introduction,” “Results,” and “Discussion.” AH wrote the section “Materials and Methods,” conducted the analysis, and edited the sections “Introduction” and “Discussion.” RD performed the data science on the trace data, organized and extracted the data, and involved in editing all the sections of the manuscript.

## Conflict of Interest

The authors declare that the research was conducted in the absence of any commercial or financial relationships that could be construed as a potential conflict of interest.

## Appendix

**TABLE A1:** Overview of number of learners that calibrated, over or underestimated on goal attainment and performance.

	**Calibration goal attainment**			**Calibration performance**		
		
	**Calibrates**	**Underestimate**	**Overestimate**	**Calibrates**	**Underestimate**	**Overestimate**
Lesson 1	11	9	14	0	19	4
Lesson 2	15	6	15	0	4	2
Lesson 3	17	10	9	2	13	7

**TABLE A2:** *Correctness of the signals*: overview of number of learners that calibrated, under or overestimated on accuracy attainment and accuracy.

	**Calibration accuracy attainment**			**Calibration actual accuracy**		
		
	**Calibrates**	**Underestimate**	**Overestimate**	**Calibrates**	**Underestimate**	**Overestimate**
Lesson 1	8	22	4	5	17	13
Lesson 2	12	14	10	2	16	17
Lesson 3	15	8	13	1	13	19
